# Task-irrelevant semantic relationship between objects and scene influence attentional allocation

**DOI:** 10.1038/s41598-024-62867-6

**Published:** 2024-06-07

**Authors:** Joseph C. Nah, George L. Malcolm, Sarah Shomstein

**Affiliations:** 1 Bose Corporation, Framingham, USA; 2 School of Psychology, University of East Anglia, Norwich, UK; 3 Department of Psychological and Brain Sciences, The George Washington University, Washington, DC, USA

**Keywords:** Psychology, Human behaviour

## Abstract

Recent behavioral evidence suggests that the semantic relationships between isolated objects can influence attentional allocation, with highly semantically related objects showing an increase in processing efficiency. This semantic influence is present even when it is task-irrelevant (i.e., when semantic information is not central to the task). However, given that objects exist within larger contexts, i.e., scenes, it is critical to understand whether the semantic relationship between a scene and its objects continuously influence attention. Here, we investigated the influence of task-irrelevant scene semantic properties on attentional allocation and the degree to which semantic relationships between scenes and objects interact. Results suggest that task-irrelevant associations between scenes and objects continuously influence attention and that this influence is directly predicted by the perceived strength of semantic associations.

## Introduction

Through experience, we learn that certain objects are likely to be present in a scene: going into an office means seeing desks, computers, and keyboards; while going into a restaurant means being surrounded by tables, plates, and utensils. Semantic information, such as knowing that a chair appears near a desk within an office, is a ubiquitous feature innate in all objects in our environment. Decades of research have demonstrated that semantic information is rapidly and automatically extracted from scenes^1–6^, and that semantic context, when relevant to the task, influences attention^7–12^. However, whether semantic context influences attention when it is not directly relevant to the task has been underspecified. Understanding whether semantic context influences attention continuously and independent of the task at hand (i.e., task-irrelevant) is central to developing predictive models of attentional allocation in any given scene or environment. If semantic information influences attention even when task-irrelevant, then it can be concluded that semantic influence is a default signal that dynamically contributes to attentional orienting^13,14^ necessitating revision of our understanding of what aspects of the scene contribute to perception.

A somewhat non-intuitive aspect of our environment is that most of the information impinging on our senses is essentially irrelevant to any particular task that we are engaged in at any moment in time^15^. Imagine waiting for the signal at a crosswalk. While the signal color, passing cars, pavement, and people around you are relevant, this information in aggregate comprises only a small subset of all information available within the environment around you. For example, the parked cars and bikes, buildings, trees, color of the sky, conversations, smells, etc., are all task-irrelevant. Is attention influenced by this omnipresent and abundant yet mostly irrelevant information?

A few recent studies provide a starting point for answering this question, showing that task-irrelevant semantic information facilitates spatial attention to individual objects that are semantically-related without any context^16–18^ and guides attention within a scene that is rich in context^19,20^. For instance, when examining a real-world scene, an object semantically unrelated to the scene can modulate gaze behavior leading to increased fixation durations or dwell time^20^. However, our everyday experience usually consists of semantically meaningful scenes containing multiple semantically informative objects^18^. As a result, each element in the scene serves to support a semantically consistent representation, where content is related to each other. Thus, an unanswered question remains—how does the relationship between individual objects and their relationship with scenes interact to influence attention when task-irrelevant?

Here, across four experiments, we systematically manipulate the semantic relationship *between objects and scenes* to directly test the degree to which task-irrelevant semantic associations interact. Experiments 1–3 investigate the influence of semantic relationship between objects and scenes. It is hypothesized that if semantic associations guide attentional selection, independent of task, the high-level information extracted from a scene will facilitate processing of semantically related objects, leading to more efficient processing of targets presented on semantically related task-irrelevant object. Importantly, we further show a direct relationship between the strength of semantic association and the magnitude of attentional facilitation (e.g., a computer mouse in an office would be processed faster than when in a bathroom). Lastly, Experiment 4 investigates how the object-to-object semantic relationship and object-to-scene semantic relationships interact, with the prediction that maximum attentional benefit will be observed when all semantic relationships are congruent, suggesting a more efficient processing of information.

## Results

### Experiment 1: Task-irrelevant semantic scene-to-object relationship influences attention

#### Accuracy

A two-way repeated measure analysis of variance (ANOVA) was conducted with scene category (office, living room, bathroom, kitchen, bedroom) and semantic relationship (semantically related, SR; semantically not related, NR) as within-subjects variables. Main effects were not significant (*F*s < 1.0, *p*s > 0.36). As hypothesized, there was a significant interaction with scene category and semantic relationship, *F*(4, 64) = 3.60, p = 0.010, ${\eta }_{p}^{2}$ = 0.184. In a follow up analysis, consistent with our hypothesis, participants were significantly more accurate (M = 90.17%) in the non-related (NR) than the semantically related (SR) condition (M = 86.83%) for the bedroom scene category (*F*(1) = 9.30, *p* = 0.008), and marginally more accurate in the SR (M = 91.54%) than the NR (M = 88.43%) condition for the bathroom category (*F*(1) = 4.38, *p* = 0.053). No other significant difference existed for the remaining three scene categories (*p*s > 0.40).

**Figure 1:**
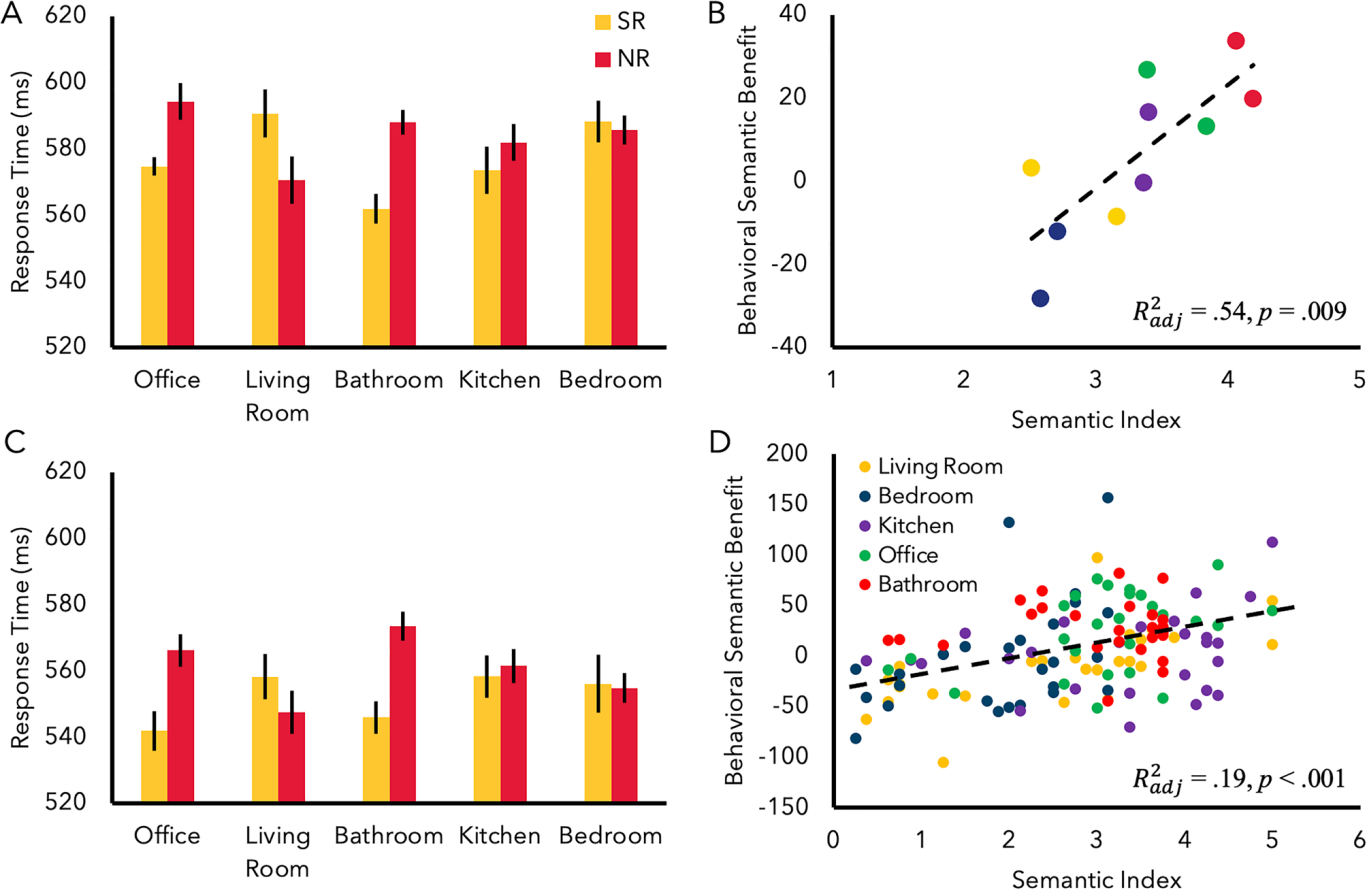
(**A**) RT results of Experiment 1. (**B**) Regression analysis for Experiment 1 indicated that the strength of semantic association significantly predicted the amount of semantic facilitation. Two data points per category represent the two semantically related objects for each scene. Error bars indicate ± 1 standard error. (**C**) RT results for Experiment 2. (**D**) Regression analysis again revealed a significant relationship between semantic index and behavioral semantic facilitation. Datapoints here represent each individual participant for each scene.

*RT.* The same ANOVA was conducted for RT, revealing a significant main effect of semantic relationship consistent with the prediction that semantic relationship influences attentional allocation was observed: overall faster response in SR (M = 577.78 ms) than NR (M = 584.11 ms) condition, *F*(1, 16) = 4.57, *p* = 0.048, = ${\eta }_{p}^{2}$0.22 (Fig. 1A). There was no significant main effect of scene category (*F* < 1.5, *p* = 0.21). There was a significant interaction with scene category and semantic relationship, *F*(4,64) = 4.91, *p* = 0.002, ${\eta }_{p}^{2}$ = 0.24 (Fig. 1A), suggesting that the semantic benefit was not uniformly observed across all categories. Simple main effects analysis revealed that participants were significantly faster at responding in SR (M = 574.67 ms) than NR (M = 594.30 ms) condition in the office (*F*(1) = 9.19, *p* = 0.008) and bathroom (SR: M = 561.89 ms; NR: M = 588.02 ms), (*F*(1) = 16.91, *p* =  < 0.001) and were marginally significantly faster at responding in NR (M = 570.64 ms) than SR (M = 590.62 ms) condition in the living room scene category (*F*(1) = 4.44, *p* = 0.051). No other simple main effects were significant (*p*s > 0.35).

#### Semantic regression

Following the significant interaction between scene category and semantic relationship, we directly tested the prediction that the semantic benefit is only as strong as the strength of semantic relationship between objects and scenes. We thus investigated whether attentional facilitation is directly dependent on the strength of association that an object has with the scene. To determine whether the strength of semantic association was predictive of the amount of semantic benefit, data from the survey were utilized. For each scene category, the average rating for all eight objects that were NR (e.g., oven mitt, alarm clock, toothpaste, etc. for office) was subtracted from the rating of a SR object (e.g., mouse for office) to calculate the semantic index, with a greater value indicating a stronger semantic association. This calculation was done for all objects used in the experiment. Then, a linear regression analysis was conducted to test whether the strength of the semantic association significantly predicted the amount of semantic facilitation in RT (Fig. 1B), defined as the difference in RT between the NR and SR condition (NR – SR; greater value indicating greater semantic benefit).

The results indicated that the semantic association was highly predictive of overall semantic facilitation, ${R}_{adj}^{2}$ = 0.54, *F*(1,8) = 11.67, *p* = 0.009. For example, objects associated with the bathroom (toothpaste and toilet paper; red data points) were strongly associated with the scene resulting in a strong semantic facilitation, while objects associated with the bedroom (clothes hanger and alarm clock; blue data points) were not strongly associated, leading to a weaker semantic facilitation.

The current results show that attentional allocation is influenced by task-irrelevant meaningful associations between a scene and objects. RT data indicate that participants were significantly faster at target identification when the target appeared on the semantically-related object than the non-related object, linking attentional allocation with semantic relatedness. This semantic effect interacted with scene category, where office and bathroom scenes yielded largest facilitation, with weaker effects in living room, kitchen, and bedroom. To test whether the semantic benefit is directly related to the strength of semantic association of object to the scene, the strength of semantic association between objects and scenes was measured. Using the derived semantic strength, a regression analysis indicated that the strength of semantic association is highly predictive of the amount of semantic facilitation: the more strongly associated an object is with a scene, relative to other objects, the stronger the amount of attentional facilitation. These results indicate that semantic relationships between scenes and objects can be processed and influence attention in an involuntary manner, demonstrating that the influence of task-irrelevant semantic information on attentional allocation can be expanded to object-scene relationships.

### Experiment 2: Strength of semantic association predicts amount of semantic influence on attention

Experiment 1 demonstrated that task-irrelevant semantic scene-object relationship influences attentional allocation by facilitating attention towards the semantically related object. To test the generalizability of this effect, multiple exemplars per scene category were utilized, and the Amazon Mechanical Turk platform was used to recruit a more diverse population. Additionally, to directly relate each individual’s semantic rating to facilitation of RTs, participants were asked to complete the semantic survey at the end of the experiment.

#### Accuracy

A two-way repeated measures ANOVA was conducted with scene category (office, living room, bathroom, kitchen, and bedroom) and semantic relationship (SR, NR) as within subjects variable. No main effects or interaction reached significance (*F*s < 2.31, *p*s > 0.06).

*RT*. Directly replicating results of Experiment 1, the same ANOVA for RT revealed a significant main effect of semantic relationship with overall faster response in SR (M = 552.13 ms) than NR (M = 560.74 ms) condition, *F*(1, 25) = 8.13, *p* = 0.009, ${\eta }_{p}^{2}$ = 0.25 (Fig. 1C),. There was no significant main effect of scene category (*F* < 1, *p* = 0.70). Additionally, there was a significant interaction with scene category and semantic relationship, *F*(4,100) = 3.88, p = 0.006, ${\eta }_{p}^{2}$ = 0.13, again replicating Experiment 1. Simple main effects analysis revealed that participants were significantly faster at responding in SR (M = 541.96 ms) than NR (M = 566.24 ms) condition in the office (*p* = 0.005) and bathroom (SR: M = 545.96 ms; NR: M = 573.53 ms), (*p* =  < 0.001) scene categories. No other simple main effects were significant (*p*s > 0.17) (Fig. 1C)i.

#### Regression analysis

A linear regression analysis was conducted to test whether the strength of the semantic association directly predicted the amount of semantic facilitation in RT. The results indicated that the semantic association was highly predictive of overall semantic facilitation, ${R}_{adj}^{2}$ = 0.19, *F*(1,128) = 30.69, *p* < 0.001 (Fig. 1D). To assess the nature of this significant relationship, participants’ semantic ratings were linearly regressed against each scene category. This revealed that semantic ratings were strongly predictive of the amount of attentional facilitation within the office category, ${R}_{adj}^{2}$ = 0.14, *F*(1,24) = 5.20, *p* = 0.032, the living room category,${R}_{adj}^{2}$ = 0.38, *F*(1,24) = 16.02, *p* < 0.001, and the bedroom category, ${R}_{adj}^{2}$ = 0.19, *F*(1,24) = 6.80, *p* = 0.015, but not for the kitchen or bathroom category (*F*s < 1.68, *p*s > 0.20).

Using multiple exemplars, Experiment 2 internally replicated and extended our findings showing that attention is influenced by the scene-object semantic relationship. This semantic benefit again interacted with scene category, such that participants showed the effect in the predicted direction in the office and bathroom scenes. A regression analysis indicated that overall, the strength of semantic association was highly predictive of the amount of semantic facilitation: a stronger object-scene association leads to greater attentional facilitation. This finding partially explains why some scenes did not yields a strong semantic bias (i.e., a weak object-to-scene association yields a weak semantic benefit).

### Experiment 3: Semantic facilitation occurs independent of statistical association

Thus far, we show strong evidence for the influence of task-irrelevant semantic scene-object association on attentional allocation. However, despite the fact that scene-object semantic relationship was not informative of the target location, one of two possible semantically-related objects always appeared in any condition (e.g., either a mouse or calculator was always present in the office). Thus, participants may have explicitly, or implicitly, learned to associate specific objects with a scene^21–23^ resulting in faster preparatory responses to the SR object. To ensure the task-irrelevant nature of the object-scene semantic relationship, a control condition where neither object was related to the scene was added, eliminating any predictive scene-object associations.

#### Accuracy

A two-way repeated measures ANOVA was conducted with scene category (office, living room, bathroom, kitchen, and bedroom) and semantic relationship (SR, NR, control) as within-subjects variable. No main effects or interaction reached significance (*F*s < 2.12, *p*s > 0.08).

**Figure 2:**
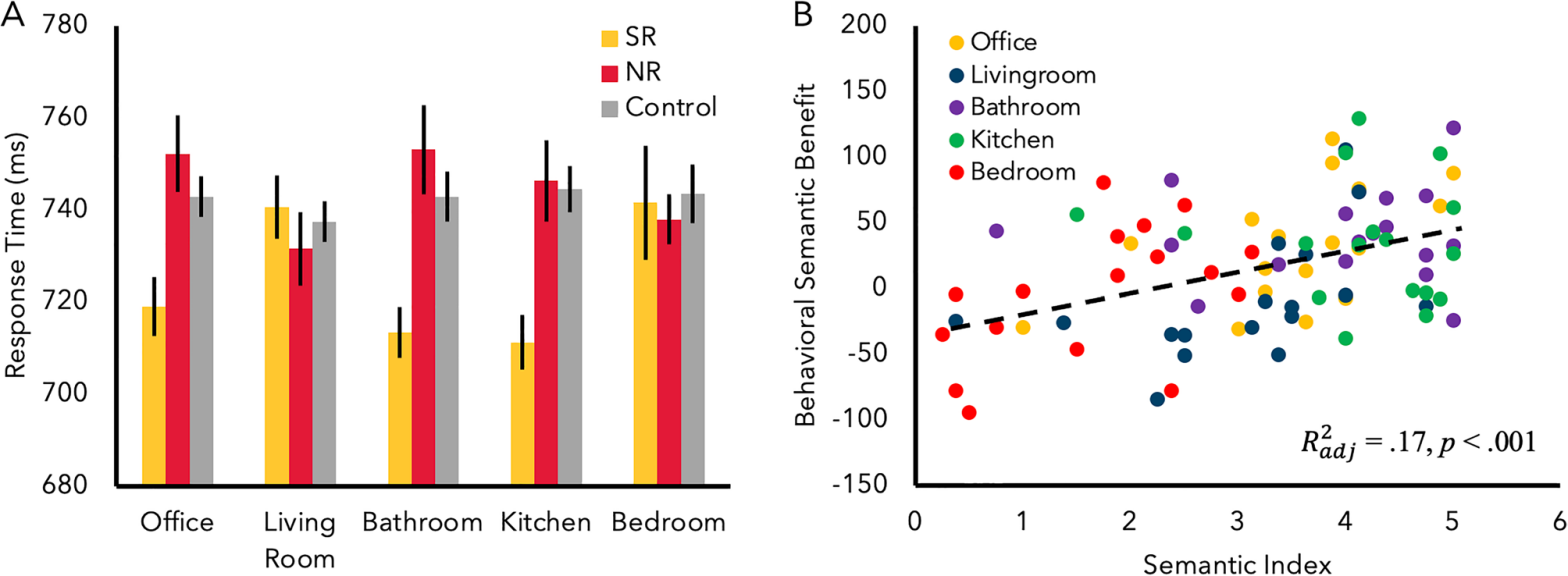
(**A**) RT data from Experiment 3. (**B**) Regression analysis for Experiment 3. Greater semantic association led to greater behavioral facilitation.

*RT*. The ANOVA for RT revealed a significant main effect of semantic relationship, *F*(2, 32) = 13.78, *p* < 0.001,${\eta }_{p}^{2}$ = 0.46 (Fig. 2A). Overall, participants were fastest in the SR (M = 711.23 ms) condition than the NR (M = 746.28 ms) and the control (M = 744.45 ms) condition (*p*s < 0.001), and there was no significant difference between the NR and control condition, (*p* = 0.622). No other main effect reached significance, (*F* < 1, *p* > 0.85). There was also a significant interaction between scene category and semantic relationship, *F*(8,128) = 2.89, *p* = 0.005, ${\eta }_{p}^{2}$ = 0.15, again replicating findings reported in Experiment 1 and 2. Simple main effects analysis revealed a significant interaction for the office, (*F*(2, 32) = 7.80, *p* = 0.002), bathroom, (*F*(2, 32) = 13.30, *p* < 0.001), and kitchen, (*F*(2, 32) = 8.11, *p* = 0.001). No other simple main effect reached significance (*p*s > 0.66). These simple main effects were further broken down, revealing that for the bathroom, participants were significantly faster at responding in the SR (M = 713.39 ms) than NR (M = 753.11 ms) (*p* < 0.001) and control (M = 742.86 ms) (*p* = 0.010) conditions, but the NR and control conditions were not significantly different (*p* = 0.29). Similarly, participants were significantly faster at responding in the SR (M = 718.94 ms) condition than the NR (M = 752.17 ms) and control (M = 742.86 ms) conditions (*p*s < 0.001) in the office, but the NR and control condition were not significantly different (*p* = 0.209). Lastly, participants were significantly faster in the SR (M = 711.23 ms) condition than the NR (M = 746.28 ms) (*p* = 0.001) and control (M = 744.45 ms) condition (*p* = 0.002) in the kitchen scene, but the NR and control conditions were not significantly different (*p* = 0.853).

#### Regression analysis

Only data from the SR and NR conditions were used in the regression analysis to calculate the behavioral semantic facilitation effect consistently with Experiments 1 and 2. A linear regression analysis (identical analysis conducted for previous Experiments) was conducted to test whether the strength of the semantic association significantly predicted the amount of semantic facilitation in RT. The results indicated that the semantic association was highly predictive of overall semantic facilitation, ${R}_{adj}^{2}$ = 0.17, *F*(1,83) = 18.50, *p* < 0.001 (Fig. 2B). To assess the nature of this significant relationship, participants’ semantic ratings were linearly regressed against each scene category. This revealed that semantic ratings were strongly predictive of the amount of attentional facilitation within the office, ${R}_{adj}^{2}$ = 0.24, *F*(1,15) = 6.03, *p* = 0.027, the living room,${R}_{adj}^{2}$ = 0.19, *F*(1,15) = 4.67, *p* = 0.047, and the bedroom,${R}_{adj}^{2}$ = 0.21, *F*(1,15) = 5.32, *p* = 0.036, but not for the kitchen or bathroom (*F*s < 1, *p*s > 0.61).

In Experiment 3, an overall effect of scene-object semantic relationship on attentional allocation was observed, even when participants were no longer able to predict presence of any particular object in a scene. As in prior experiments, faster performance was observed for targets appearing on semantically-related objects than on non-related or control objects, demonstrating that the semantically-related object is being processed preferentially. Noticeably, there was no significant difference between the NR and control condition, suggesting that the task-irrelevant semantic association between an object and a scene facilitates attentional allocation rather than inhibits non-related information. Lastly, a linear regression analysis replicated the findings of the previous two experiments, such that the strength of semantic relationship predicted the amount of behavioral semantic facilitation across all scenes as well as separately in the office, living room, and bedroom scene categories. Again, there was no relationship between the strength of semantic relationship and behavioral facilitation in the bathroom and kitchen, replicating the findings of the previous experiment.

### Experiment 4: Task-irrelevant semantic scene-object relationship interacts with object-object relationship

While Experiments 1–3 established the effect of task-irrelevant semantic relationship between objects and scene on attention, scenes are comprised of multiple objects forming even more relationships with one another^24,25^. Considering that the semantic relationship between objects can influence attention in both task-relevant and irrelevant situations, it is important to understand how the object-to-object relationship interacts with object-to-scene relationship to influence attention. In Experiment 4 the degree to which object-object relationship interacts with the semantic relationship between object and scene was examined. We hypothesized that the greatest attentional facilitation would occur when both the object-object and object-scene relationships are related, while the least amount of facilitation would be observed when neither were related. Note that as in prior experiments objects and scenes remain task-irrelevant.

#### Accuracy

A three-way repeated measures ANOVA with scene category (office, living room, bathroom, kitchen, bedroom), scene relationship (scene SR, scene NR) and object relationship (object SR, object NR) was conducted for accuracy and RT. No interaction or main effect reached significance for accuracy (*F*s < 2.44, *p*s > 0.05).

#### RT

**Figure 3:**
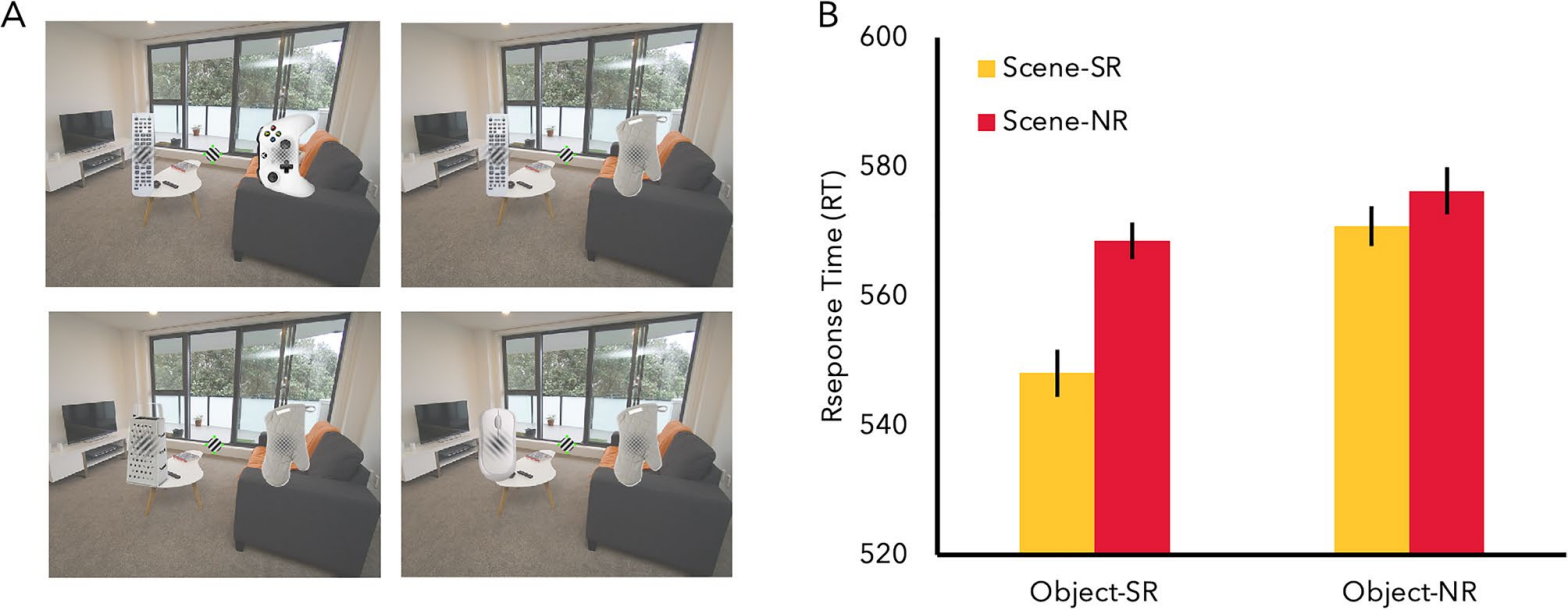
(**A**) Example of all 4 possible experimental conditions. From top left in clockwise order, object-SR & scene-SR, object-NR & scene-SR, object-NR & scene-NR, object-SR & Scene-NR (**B**) RT Results of Experiment 4. (**C**) Regression analysis for Experiment 4 indicated that the strength of semantic association again significantly predicted the amount of semantic facilitation.

The ANOVA for RT revealed significant main effects of object relationship (F(1, 16) = 15.26, p = 0.001, ${\eta }_{p}^{2}$ = 0.49) and scene relationship, (F(1, 16) = 23.42, p < 0.001, ${\eta }_{p}^{2}$ = 0.59) with significantly faster performance when both objects were semantically related (object-SR condition, M = 560 ms) than when the objects were not related (object-NR condition, M = 572.74 ms), and faster performance when the object was semantically related to the scene (scene-SR, M = 558.53 ms) than when not (scene-NR, M = 574.21 ms). The main effect for scene category did not reach significance (*F* = 2.04, p = 0.099). There was a significant two-way interaction between scene relationship and object relationship, (*F*(1, 16) = 6.26, *p* = 0.024, ${\eta }_{p}^{2}$ = 0.28) (Fig. 3B). A simple main effect analysis revealed that the interaction was driven by significantly faster responses in the object-SR condition (M = 548.33 ms) than the object-NR condition (M = 568.74 ms) in the scene-SR condition (p < 0.001). Thus, participants were fastest in performing the task when both the object on which the target appears is semantically related to both the other object and the scene category. There was no difference between the object-SR and object-NR condition in the scene-NR condition (*p* = 0.299). There was also an interaction between scene category and object relationship (*F*(4, 64) = 4.71, *p* = 0.002, ${\eta }_{p}^{2}$ = 0.23), with faster responses to the object-SR condition than object-NR condition for office (object-SR: M = 555.52 ms; object-NR: M = 583.51 ms) (p = 0.001), bathroom (object-SR: M = 548.82 ms; object-NR: M = 572.82 ms) (p = 0.008), and kitchen (object-SR: M = 567.23 ms; object-NR: M = 588.59 ms) categories (p = 0.001). There was no significant difference in any other scene categories (p > 0.25). There was also a significant interaction between scene category and scene relationship (*F*(4, 64) = 6.31, *p* < 0.001, ${\eta }_{p}^{2}$ = 0.28), with faster responses to the scene-SR condition than scene-NR condition for office (scene-SR: M = 547.84 ms; scene-NR: M = 591.19 ms) (p < 0.001), bathroom (scene-SR: M = 546.26 ms; scene-NR: M = 575.38 ms) (p < 0.001), and kitchen categories (scene-SR: M = 565.54 ms; scene-NR: M = 590.28 ms) (p = 0.013). There was no significant difference in any other scene categories (p > 0.31).

#### Semantic regression

Using the strength of semantic association values for all 10 objects obtained in Experiment 1, a linear regression analysis was conducted to test whether the strength of the semantic association significantly predicted the amount of semantic facilitation in RT (Fig. 3C). The results indicated that the semantic association was highly predictive of overall semantic facilitation, ${R}_{adj}^{2}$ = 0.59, *F*(1,8) = 14.14, *p* = 0.006. Thus, as seen consistently across previous experiments, objects that were rated as strongly associated with a scene showed the greatest behavioral facilitation while objects that were rated as being weakly associated showed the least facilitation.

Experiment 4 investigated the degree to which object-object semantic relationship interacts with scene-object semantic relationship to understand how different types of high-level associations interact to influence attentional allocation. The results replicated previous findings: participants’ performance was facilitated when the target appeared on top of the semantically related object. More importantly, there was an interaction between object-object relationship and object-scene relationship, such that the greatest facilitation was when both the objects were related to each other as well as the scene. Thus, maximum benefit was seen when everything was related to one another, suggesting a more efficient processing of scene information.

## Discussion

When viewing scenes, object and semantic information influences attentional guidance far more than saliency information alone^15,26–28^. While the focus has traditionally been on situations in which semantic information is relevant to the task^7–10,29^, semantic information is a continuous element of the real-world and thus its influence might extend beyond task-relevance. Accordingly, a growing literature argues that high-level information from real-world scenes is processed automatically and can impinge on cognitive processes^19,20^ and guide attention^16,30^. However, a question that remains unaddressed is whether the semantic information of a scene as well its relationship with an object can influence attention. Considering that objects are fundamental to a scene and contribute to scene perception^24,31,32^, it is important to understand how the task-irrelevant semantic relationship between an object and a scene contributes to attentional allocation.

Across four experiments, we demonstrated that task-irrelevant, scene-object semantic associations directly bias attention. Experiments 1–2 showed that a scene’s semantic properties bias attention towards the semantically related object and that the amount of this facilitation is directly driven by the strength of object-scene association. Experiment 3 established that the observed attentional benefit is not a result of learned associations, but of task-irrelevant semantic associations. Lastly, Experiment 4 tested the degree to which semantic relationship between an object and a scene interacts with the semantic association between two objects, demonstrating that attentional facilitation was greatest when all relationships were congruent to the scene category.

While there is evidence implicating obligatorily processed semantic information influencing attention, research has largely focused on isolating the semantic relationship, such as examining the relationship between a few objects^16,17^ or the effect of overall meaning within a scene^30^. This isolation allows a more controlled investigation, but it also restricts understanding of the dynamics of how various semantic relationships interact in the real-world scenes. For instance, when examining task-irrelevant semantic relationships between two, maximum three, real-world objects^16,17^, attention is biased towards the semantically related object while other studies have shown that inconsistent information may result in longer fixations, but does not necessarily capture attention^33^. Here, we provide evidence that the semantic properties of scenes automatically facilitate attention towards semantically related objects regardless of its task-relevance. This attentional benefit also scaled with the strength of semantic association, with greater behavioral benefit for stronger object-scene semantic associations. Thus, the intrusive influence that semantic information has on attentional allocation does not stop with objects, but can be extended to the semantic association between objects and scene.

## Methods

### Data analysis

All participants with an average accuracy rate of less than 80% were removed from the analysis due to lack of attention. For each participant, all RT less than 200 ms and greater than 1500 ms were removed from the analysis as anticipatory responses and attention lapses respectively (1.12, 0.9, 2.07, and 1.38% of trials were removed in Experiments 1, 2, 3, and 4 respectively). Response time (RT) and accuracy data for each experiment were analyzed using repeated-measures analysis of variance (ANOVA). Only correct trials were used to calculate mean RT for each participant.

### Participants

A power analysis was conducted using the G*Power program^34^ to demonstrate adequate power. Using an effect size (${\eta }_{p}^{2}$ = 0.208) and alpha level (0.05) from a previous study^16^, the power analysis revealed that a sample size of n = 16 was sufficient to achieve enough power (0.80). Therefore, for all experiments, at least 16 participants were recruited. All participants reported normal or corrected-to-normal vision and were naïve to the purpose of the experiment. All experimental procedures were approved by The George Washington Institutional Review Board (IRB) and all methods in this experiment were performed in accordance with the relevant guidelines and regulations.

In Experiment 1, 23 participants were recruited from The George Washington University and 7 were excluded from the final analysis for failing to meet the 80% criteria (average age: 19, 3 male). A separate set of participants from The George Washington University (n = 31, average age: 19.74, 9 male) participated in the online survey. In Experiment 2, 29 participants located within the US were recruited from Amazon Mechanical Turk in exchange for monetary compensation. Three participants were excluded from the final analysis based on the accuracy criteria, leaving a total of 26 participants (average age: 39.89, 13 females). In Experiment 3, 23 participants were recruited from The George Washington University and 6 were excluded from the final analysis, leaving a total of 17 participants (average age: 19, 17 females). In Experiment 4, 22 participants (average age: 19, 15 females) were recruited from The George Washington University and 5 were excluded from the final analysis, leaving a total of 17 participants. All participants gave written informed consent and were provided with course credit (Experiment 1, 3, 4) or monetary compensation (Experiment 2).

### Apparatus and stimuli

**Figure 4:**
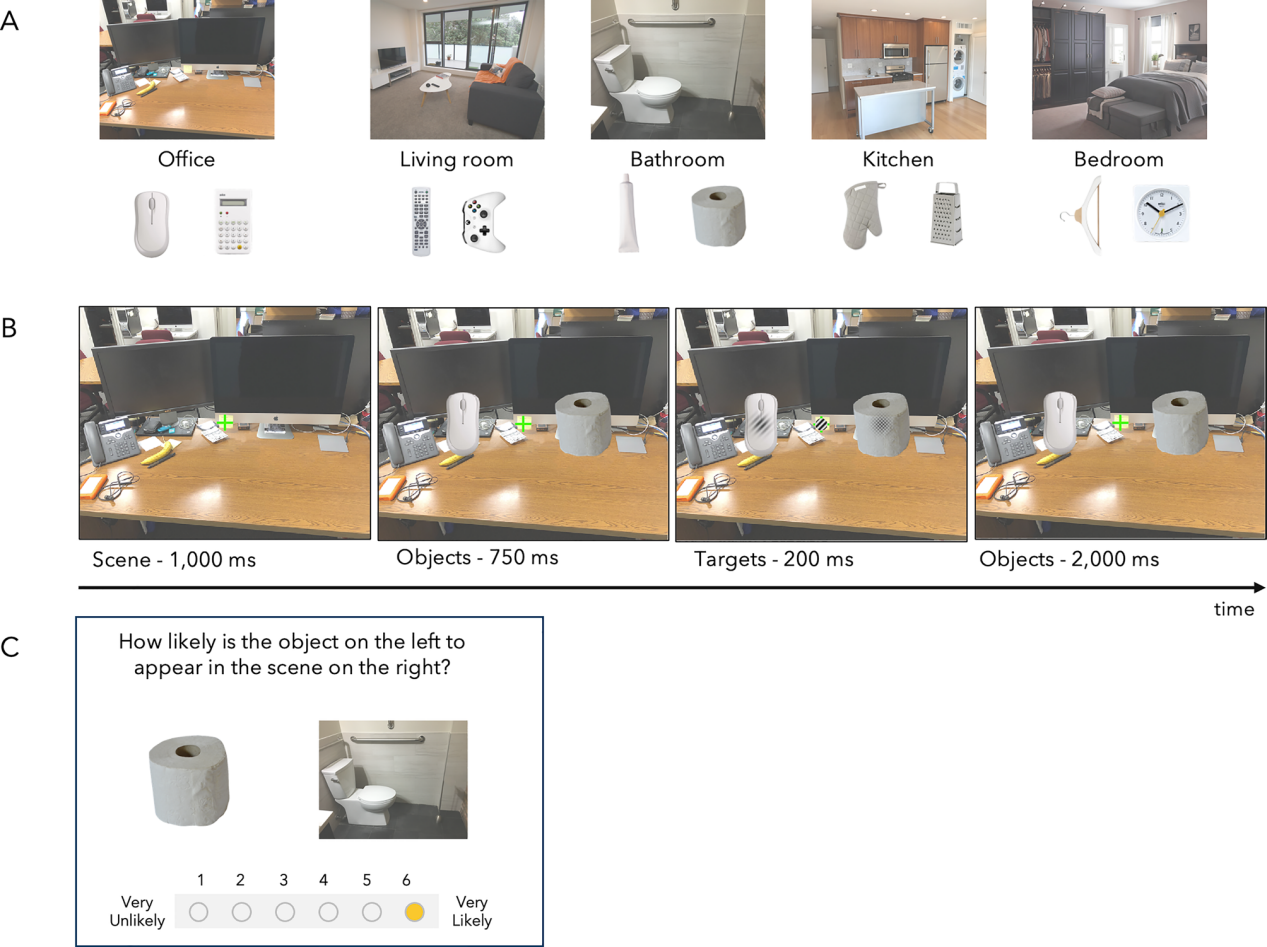
(**A**) Full set of stimuli used in Experiment 1. Objects below each scene category are the two objects designated as semantically related. (**B**) Experimental paradigm of Experiment 1—Participants’ task was to maintain fixation and report whether the orientation of the center Gabor matched the orientation of the Gabor on either one of the objects. Please refer to the Methods section for more details. (**C**) Example of survey used to rate strength of semantic association between scenes and objects.

All in-lab experiments took place in a dimly illuminated room with a 19″ Dell 1908FP color liquid crystal display monitor (60 Hz) placed approximately 60 cm from the participant. The experiment was conducted using Python 2.7 and generated using the PsychoPy library^35,36^. The scene stimuli were 16° × 12.8° in size and the object stimuli were 3° in height with width varying from 1° to 2°. A total of 5 scene images were used in the experiment (3 photographed by the author and 2 found using Google search) and were partially desaturated to reduce potential low-level biasing and make the objects more visible (Fig. 4A). The 5 scene images were of different indoor categories (office, living room, bathroom, kitchen, bedroom). A total of 10 objects were used in the experiment with 2 designated as semantically related to a specific scene category (e.g., office with mouse and calculator, bathroom with toilet paper and toothpaste). Images of the objects were found online (using Google search).

The online experiment (Experiment 2) was conducted on Amazon Mechanical Turk using psiTurk^37^ and on each participants’ computer outside of the laboratory. A total of 8 exemplars were used for each scene category for a total of 40 unique scene images. All stimuli were selected from Google Images as well as in-house. All other elements of the experiment were identical to the in-lab experiments.

### Design and procedure

Each trial began with a fixation cross presented on a gray screen for 500 ms. Afterwards, a scene image was presented for 1,000 ms with participants being instructed to maintain fixation. This was followed by the onset of a peripheral object pair, 3.5° from either side of fixation (Fig. 4B) for 750 ms. Critically, one of the objects was always semantically related (SR) to the scene while the other object was not related (NR). After the objects were displayed for 750 ms, two target Gabor patches (one smaller than the other) and distractor was presented. The smaller Gabor patch (0.3°) was always presented in the middle of the scene, on top of the fixation cross. The larger Gabor patch (1.5°) was presented in the middle of one of the two objects and a checkerboard distractor (1.5°) appeared on the other object. The semantic relationship of the object on which the target Gabor appeared defined the experimental condition (e.g., SR = target on a semantically related object, NR = target on non-related object object). The Gabor patches were oriented 45° either to the left or right. Participants were instructed to maintain fixation and report whether the Gabor patches matched in orientation with a key press. Crucially, the larger Gabor patch appeared equally on both the SR and NR object, rendering the scene-object semantic association task-irrelevant. The targets/distractor were presented for 200 ms, after which participants had 2,000 ms to respond. The brief presentation of the targets and distractor as well as the spatial distance between the fixation and objects required participants to maintain fixation to accurately perform the task, controlling for any potential eye movements. Correct trials were separated by a 500 ms intertrial interval (ITI), and incorrect trials were indicated by a red fixation cross and a 1,000 ms ITI. After a brief practice, participants were presented with the experiment.

In Experiment 1, participants performed a total of 640 trials (10 blocks of 64 trials) in which the stimuli appeared in random order. An online survey was conducted using Google Forms (on a separate set of participants), to assess the strength of semantic association between the scene and the corresponding objects used in the experiment. In this survey, participants were presented with an image of an object and a scene (Fig. 4C) and asked to rate how likely the object would appear in the scene on a scale of 1 (not very likely)–6 (very likely). These values were used to calculate the strength of semantic association to determine whether, as predicted, the strength of semantic relationship between an object and a scene can be used to predict the strength of semantic benefit.

In Experiment 2, participants performed a total of 320 trials (5 blocks of 64 trials). The number of trials were halved to ensure participants would focus and finish the experiment within 30 min. Participants also took part in the semantic rating survey at the end of the experiment. Participants were presented with an image of an object and were asked to rate how likely the object would appear in one of the five scene categories on a scale of 1 (not very likely)–6 (very likely). For each scene category, the average rating for all eight objects that were NR for a certain scene was subtracted from the average rating of the SR objects to calculate the semantic index, indicating the strength of the semantic association.

Experiment 3 was identical to Experiment 1 except for the new control condition. The control condition consisted of trials in which, for each scene category, two objects from a separate category (e.g., toilet paper and toothpaste for office) were designated as the two objects appearing with NR objects (i.e., neither of the objects in the control condition were semantically related to each other nor the scene). With the addition of the control condition, participants were now equally likely to see trials in which an object was related to the scene and trials in which neither was related. 50% of all trials were the control condition with the remaining half equally split between the SR and NR condition.

Experiment 4 utilized a 2 (object-object relationship: object-SR, object-NR) × 2 (object-scene relationship: scene-SR, scene-NR). The condition was defined based on the semantic relationship of the object on which the target appeared (Fig. 3A). Thus, an object-SR condition would mean that the object with the target Gabor patch would be semantically related to the other object and the scene-SR condition would mean that the scene would be semantically related to the object with the target Gabor patch. As with all previous experiments, the target could appear on the objects with equal probability, rendering semantic associations task-irrelevant. All other aspects of the experiment was identical to Experiment 1.

## Data Availability

Data from this study are available at Open Science Framework (OSF: https://osf.io/mzvsp/).

## References

[1] Potter MC (1976). Short-term conceptual memory for pictures. J. Exp. Psychol. Hum. Learn..

[2] Potter MC, Levy EI (1969). Recognition memory for a rapid sequence of pictures. J. Exp. Psychol..

[3] Thorpe S, Fize D, Marlot C (1996). Speed of processing in the human visual system. Nature.

[4] Potter MC (2014). Detecting meaning in RSVP at 13 ms per picture. Attent. Percept. Psychophys..

[5] Potter MC (2012). Recognition and memory for briefly presented scenes. Front. Psychol..

[6] Fei-Fei L (2007). What do we perceive in a glance of a real-world scene?. J. Vis..

[7] Moores E, Laiti L, Chelazzi L (2003). Associative knowledge controls deployment of visual selective attention. Nat. Neurosci..

[8] Castelhano MS, Witherspoon RL (2016). How you use it matters: Object function guides attention during visual search in scenes. Psychol. Sci..

[9] Castelhano MS, Heaven C (2010). The relative contribution of scene context and target features to visual search in scenes. Attent. Percept. Psychophys..

[10] Mack SC, Eckstein MP (2011). Object co-occurrence serves as a contextual cue to guide and facilitate visual search in a natural viewing environment. J. Vis..

[11] Spotorno S, Malcolm GL, Tatler BW (2014). How context information and target information guide the eyes from the first epoch of search in real-world scenes. J. Vis..

[12] Torralba A (2006). Contextual guidance of eye movements and attention in real-world scenes: The role of global features in object search. Psychol. Rev..

[13] Todd RM, Manaligod MGM (2017). Implicit guidance of attention: The priority state space framework. Cortex.

[14] Shomstein S, Gottlieb J (2016). Spatial and non-spatial aspects of visual attention: Interactive cognitive mechanisms and neural underpinnings. Neuropsychologia.

[15] Shomstein S, Malcolm GL, Nah JC (2019). Intrusive Effects of Task-irrelevant information on visual selective attention: Semantics and size. Curr. Opin. Psychol..

[16] Malcolm GL, Rattinger M, Shomstein S (2016). Intrusive effects of semantic information on visual selective attention. Atten. Percept. Psychophys..

[17] Nah JC, Malcolm GL, Shomstein S (2021). Task-irrelevant semantic properties of objects impinge on sensory representations within the early visual cortex. Cereb. Cortex Commun..

[18] Nah JC, Geng JJ (2022). Thematic object pairs produce stronger and faster grouping than taxonomic pairs. J. Exp. Psychol. Hum. Percept. Perform..

[19] Greene MR, Fei-Fei L (2014). Visual categorization is automatic and obligatory: Evidence from Stroop-like paradigm. J. Vis..

[20] Cornelissen TH, Võ ML (2017). Stuck on semantics: Processing of irrelevant object-scene inconsistencies modulates ongoing gaze behavior. Atten. Percept. Psychophys..

[21] Chun MM, Jiang Y (1998). Contextual cueing: Implicit learning and memory of visual context guides spatial attention. Cogn. Psychol..

[22] Zhao J, Al-Aidroos N, Turk-Browne NB (2013). Attention is spontaneously biased toward regularities. Psychol. Sci..

[23] Zhao L (2014). Visual statistical learning can drive object-based attentional selection. Attent. Percept. Psychophys..

[24] Malcolm GL, Groen II, Baker CI (2016). Making sense of real-world scenes. Trends Cogn. Sci..

[25] Wu CC, Wick FA, Pomplun M (2014). Guidance of visual attention by semantic information in real-world scenes. Front. Psychol..

[26] Henderson JM, Malcolm GL, Schandl C (2009). Searching in the dark: Cognitive relevance drives attention in real-world scenes. Psychon. Bull. Rev..

[27] Henderson JM, Hayes TR (2017). Meaning-based guidance of attention in scenes as revealed by meaning maps. Nat. Hum. Behav..

[28] Xu J (2014). Predicting human gaze beyond pixels. J. Vis..

[29] Belke E (2008). Top-down effects of semantic knowledge in visual search are modulated by cognitive but not perceptual load. Percept. Psychophys..

[30] Peacock CE, Hayes TR, Henderson JM (2018). Meaning guides attention during scene viewing, even when it is irrelevant. Atten. Percept. Psychophys..

[31] MacEvoy SP, Epstein RA (2011). Constructing scenes from objects in human occipitotemporal cortex. Nat. Neurosci..

[32] Gagne CR, MacEvoy SP (2014). Do simultaneously viewed objects influence scene recognition individually or as groups? Two perceptual studies. PLoS ONE.

[33] Võ ML, Henderson JM (2011). Object-scene inconsistencies do not capture gaze: Evidence from the flash-preview moving-window paradigm. Attent. Percept. Psychophys..

[34] Faul F (2007). G*Power 3: A flexible statistical power analysis program for the social, behavioral, and biomedical sciences. Behav. Res. Methods.

[35] Peirce JW (2009). Generating stimuli for neuroscience using PsychoPy. Front. Neuroinform..

[36] Peirce JW (2007). PsychoPy–Psychophysics software in Python. J. Neurosci. Methods.

[37] Gureckis TM (2016). psiTurk: An open-source framework for conducting replicable behavioral experiments online. Behav. Res. Methods.

